# Supplementary material for: GroEL actively stimulates folding of the endogenous substrate protein PepQ
Source: Nat Commun. 2017 Jun 30;8:15934. doi: 10.1038/ncomms15934 (PMC5497066; doi:10.1038/ncomms15934)
Supplement: Supplementary Information [file ncomms15934-s1.pdf]

Type of file: PDF
Size of file: 0 KB
Title of file for HTML: Supplementary Information
Description: Supplementary figures and supplementary references.

Type of file: PDF
Size of file: 0 KB
Title of file for HTML: Peer review file
Description:

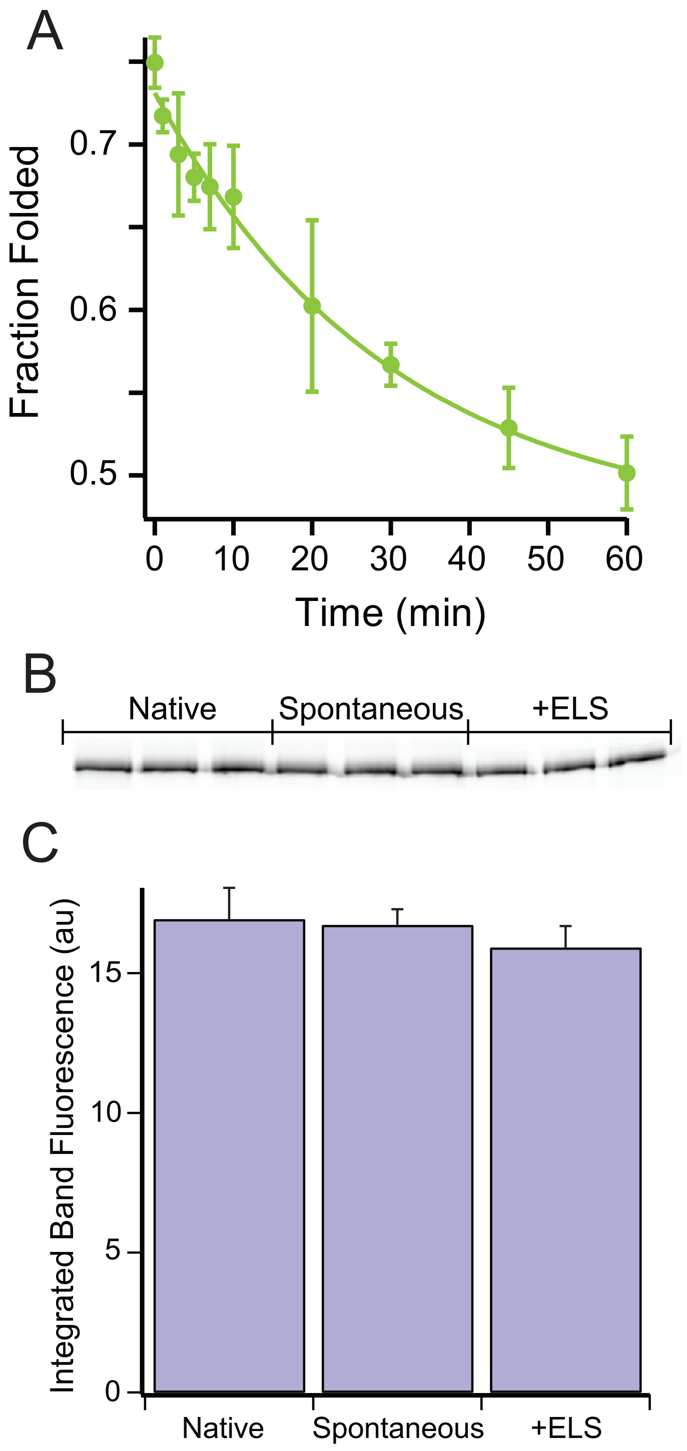

Supplementary Figure 1. PepQ slowly and spontaneously populates a misfolded state that cannot be rescued by GroEL, but which remains completely soluble. (A) The rate at which PepQ becomes refractory to refolding by GroEL was measured by

examining the loss of recoverable enzymatic activity over time. PepQ was denatured in acid-urea, diluted directly into buffer (100 nM) and then incubated at 23 °C. At the indicated times, samples were removed and mixed with an equal volume of a solution containing GroEL (400 nM) and GroES (800 nM), followed by addition of ATP (2mM). Each GroEL-supplemented sample was incubated for 60 min to permit assisted folding to reach completion; the total amount of native PepQ was determined by enzymatic assay. The observed final PepQ yields were fit to a single-exponential rate law (solid line), resulting in an observed rate of 0.033 ± 0.006 min^{-1}. Error bars show the standard deviation of three independent experiments. (B) The amount of PepQ in solution during the folding reaction was measured to determine if the decreased yield in spontaneous folding results from loss to the microcentrifuge tube surface. PepQ-24F was denatured in acid-urea and then diluted into either buffer alone (100 nM; *Spontaneous*) or buffer containing GroEL (200 nM). For the GroEL sample, GroES (400 nM) and ATP (2 mM) was added to initiate folding (+*ELS*). Native PepQ-24F was also diluted into buffer (100 nM; *Native*). Samples were taken after 60 minutes and analyzed by SDS-PAGE with fluorescent gel scanning. Each lane shows an independent folding experiment. (C) Gel bands from (B) were quantified with ImageJ. Error bars show the standard deviation of three independent experiments.

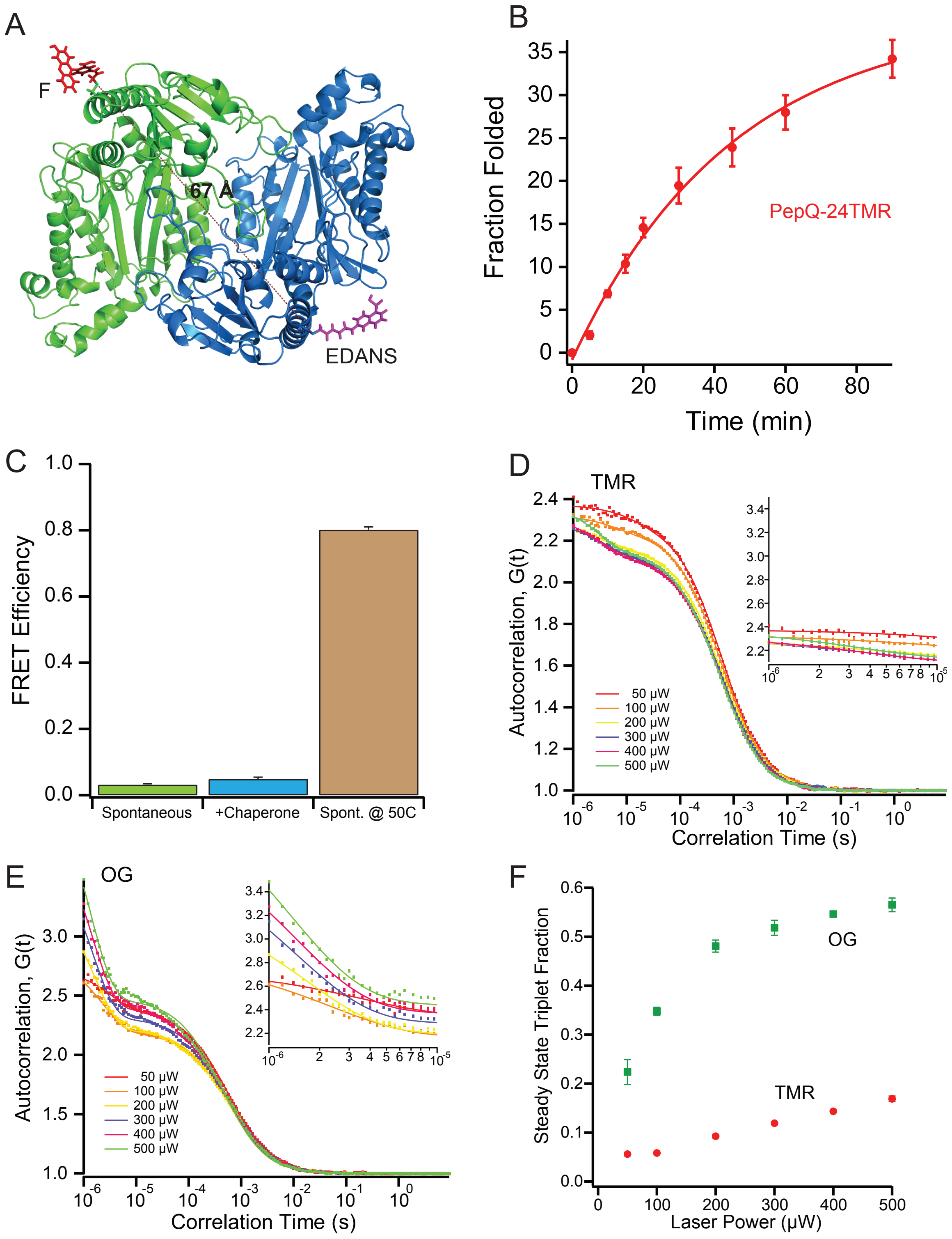

Supplementary Figure 2. Characterization of PepQ variants labeled with fluorescent dyes. (A) Structure of the PepQ dimer [1] (PDB ID: 4QR8) showing the

position of an engineered Cys residue at position 24 that was used for the attachment of the exogenous fluorescent dyes like EDANS and fluorescein (*F*). For the purpose of illustration, dye structures were modeled into the PepQ structure using PyMol. While the dye orientations shown should only be considered approximate, the distance between the Cys sulfur atoms across the native dimer interface should be ~ 67 Å. (B) Spontaneous folding of PepQ-24TMR folding was monitored by the recovery of enzymatic activity. PepQ was denatured in acid-urea and then diluted into buffer (100 nM). Data were fit to a single-exponential rate law (solid lines), resulting in observed folding rates of 0.022 ± 0.004 min^{-1} for the spontaneous reaction. Error bars show the standard deviation of three independent experiments. Folding of PepQ-24C labeled with the other exogenous fluorescent probes used in this study were all very similar. (C) PepQ aggregation was monitored with intermolecular FRET using two different, denatured populations of labeled PepQ. For the donor sample, PepQ was labeled at position 24 with IAEDANS (PepQ-24ED). The acceptor sample was created by labeling position 24 with fluorescein (PepQ-24F). Acid-urea denatured, donor-labeled PepQ (50 nM) and either denatured, unlabeled PepQ (50 nM) or denatured, acceptor-labeled PepQ (50 nM) were mixed together and diluted into buffer alone at 23 °C (*green*), 55 °C (*brown*), or into buffer containing GroEL (200 nM) at 23 °C, followed by the addition of GroES (400 nM) and ATP (2 mM; *blue*). The observed donor-side, steady state FRET efficiency was calculated from the donor fluorescence emission spectra of matched donor-only and donor-acceptor samples. Error bars show the standard deviation of three experimental replicates. In the absence of aggregation, efficient folding should result in no detectable FRET from a mixed PepQ sample, assuming a folding efficiency

of 80-90% (Figure 1B), and noting that the distance between labeled positions is at least 20 Å greater than the Förster distance typically observed with this pair of probes [2] (Figure S2A). The FRET efficiency observed following productive folding with GroEL is 4.9 ± 0.5%, while for spontaneous folding the observed FRET efficiency is 3.2 ± 0.5%. PepQ can be forced to form aggregates that are easily detectable by FRET (80 ± 0.8% efficiency) by raising the sample temperature to 50 °C during the initial dilution of PepQ from denaturant. (D-F) The propensity of the fluorescent probes, TMR and OG, to convert to the triple state was measured through changes in the FCS curve of PepQ labeled with each probe as a function of excitation power. The autocorrelation of native PepQ-24TMR (D) or PepQ-25OG (E) (1 nM dimer) was measured at different input laser powers (50-500 uW; 488 nm for OG and 561 nm for TMR). In each case, the FCS curve was acquired three times at each power setting; one representative trace for each power setting is shown for each dye. Data were fit using an autocorrelation function incorporating a term for triplet state formation by the dye [3,4]. (F) The steady state triplet fraction of each dye from the fits in (D) and (E) was plotted against the corresponding laser power. Error bars represent the average of three traces.

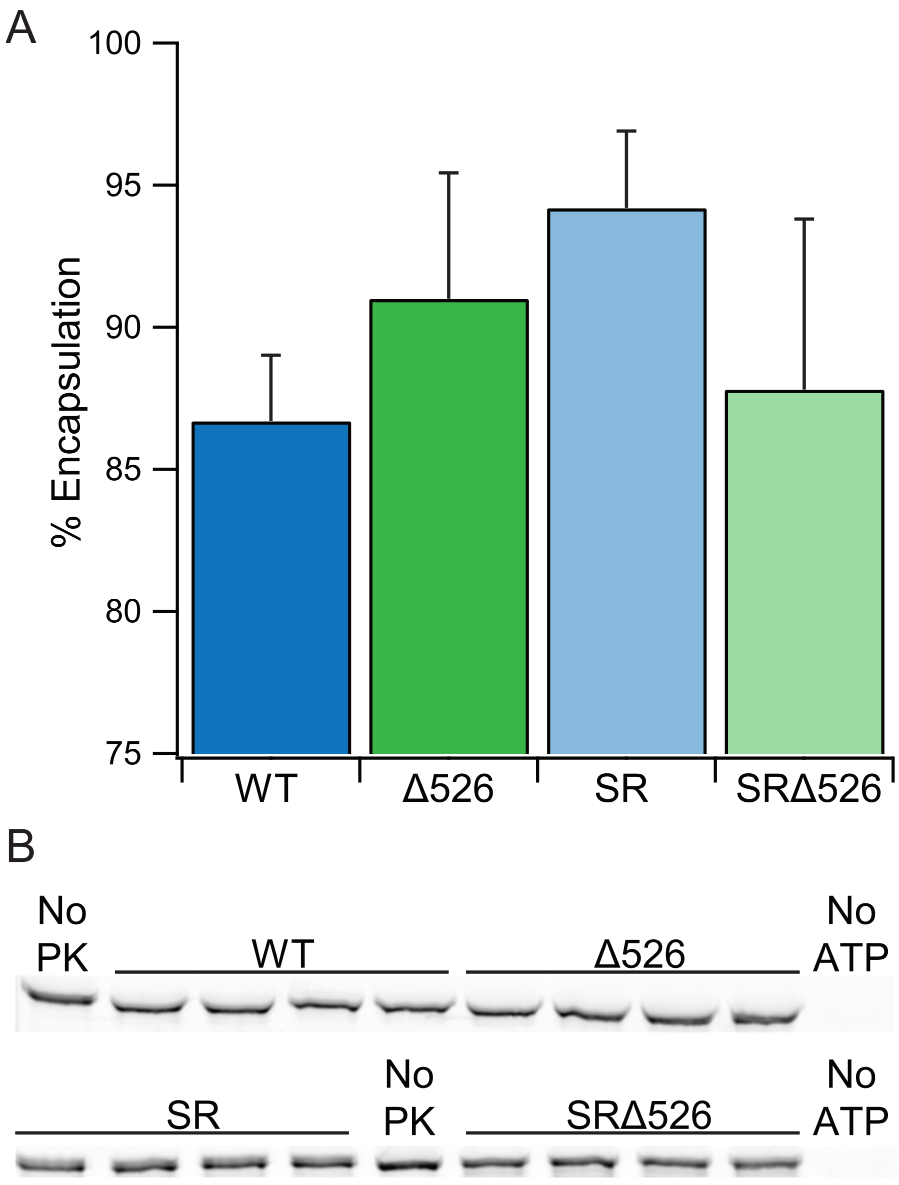

Supplementary Figure 3. Reduced folding rate in GroEL truncation mutants is not caused by a severe encapsulation deficiency. (A) PepQ-24F (100nM) was bound to wild-type or ∆526 GroEL-ES-ADP bullets (200nM) or full-length single ring GroEL (SR) or ∆526 single ring GroEL (SR∆526, 300nM) and supplemented with GroES (600nM). A single turnover was initiated by the addition of ATP (2mM) followed by quenching with

hexokinase and glucose after 10 seconds. Un-encapsulated PepQ was digested with Proteinase K (0.5 g/mL). Samples were run on SDS-PAGE and analyzed by fluorescent gel scanning, seen in (B). Samples were normalized to undigested controls. Error bars show the deviation of n = 4 independent samples.

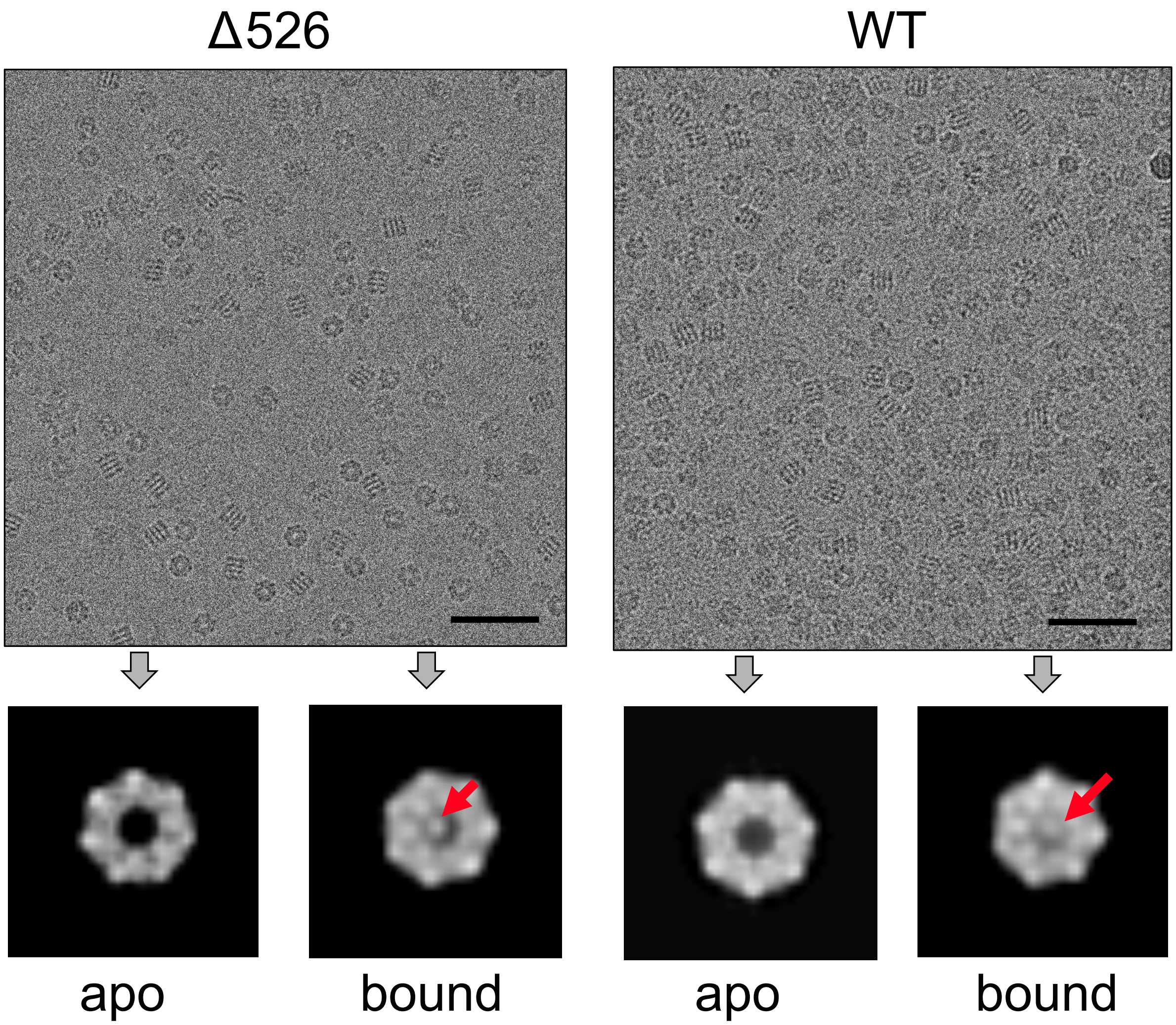

Supplementary Figure 4. Reference-free 2D class-averages of ∆526 GroEL and wild type GroEL tetradecamers incubated with nonnative PepQ. Panel A and B show representative, raw data micrographs for ∆526 GroEL (*∆526*) and wild type GroEL (*WT*) incubated with non-native PepQ. Panels C, D, E, F show the reference-free 2D classifications of tetradecamers that either did (*bound*), or did not (*apo*), bind PepQ. The black scale bars denote 100nm. The apo states of both wild type and ∆526 tetradecamers display perfect seven fold symmetry, with an empty central cavity. By contrast, the PepQ-bound states of both tetradecamers show a very clear extra density inside the central cavity, highlighted by the red arrows.

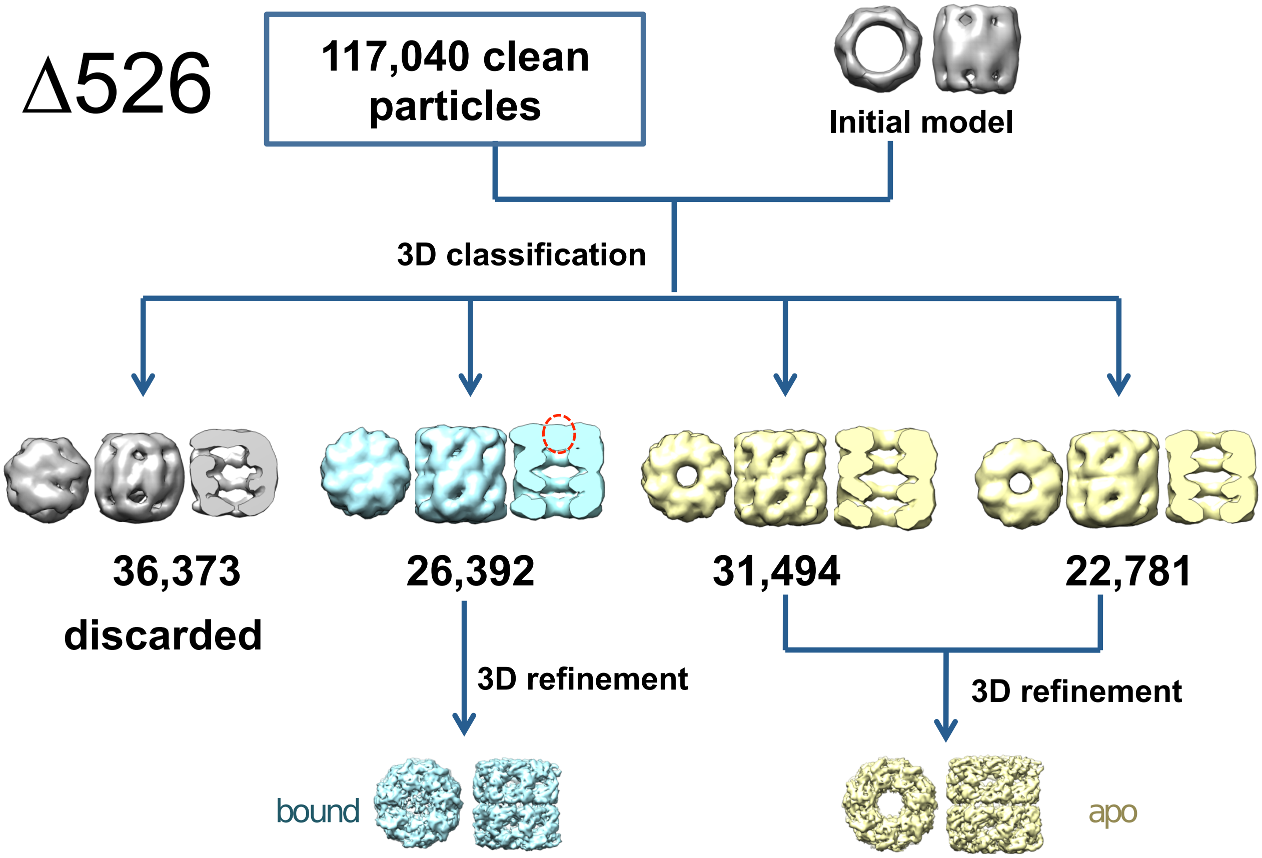

Supplementary Figure 5. Image processing for the wild type GroEL complexes. Two runs of reference free 2D classification resulted in 170,639 clean particles. The apo state of *E.coli* GroEL was used as an initial model to generate four 3D classes with C1 symmetry. The number of particles in each 3D class is shown. In Class 2, a thick extra density was readily visible in the central cavity of a well-shaped tetradecamer, which is highlighted by the dashed red circle. Particles of this class were used to generate the refined density map of the PepQ-bound wild type GroEL complex at 8.3 Å resolution. Class 3 and 4 possess particle shapes consistent with an empty GroEL tetradecamer and were used to generate the refined density map of the apo Δ526 GroEL at 7.9 Å

resolution. Because of its anomalous particle shape, Class 1 was discarded. The initial refinement model employed was derived from the crystal structure of *E. coli* GroEL (PDB ID: 4HEL), blurred to a resolution of 40 Å.

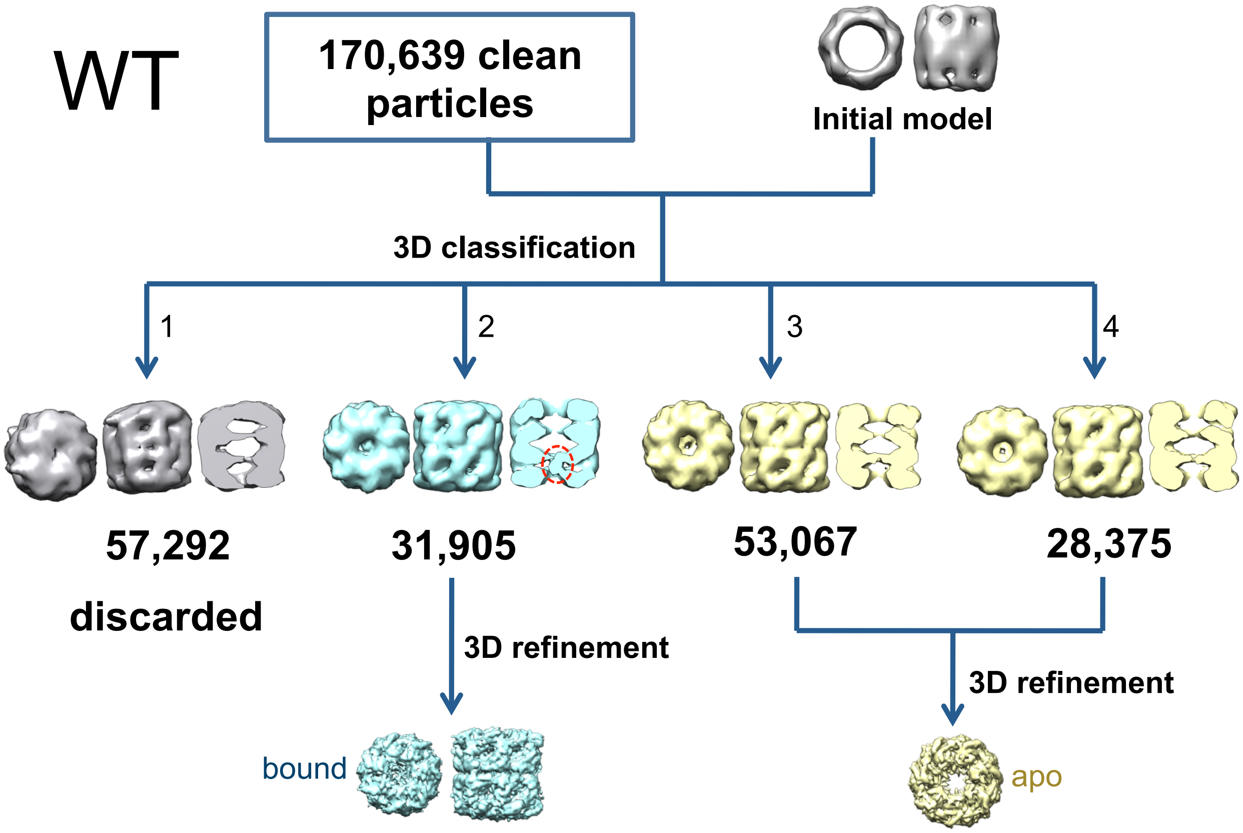

Supplementary Figure 6. Image processing for the Δ526 complexes. Two runs of reference free 2D classification resulted in 117,040 clean particles. The apo state of *E.coli* GroEL was used as an initial model to generate four 3D classes with C1 symmetry. The number of particles in each 3D class is shown. In Class 2, a thick extra density was readily visible in the central cavity of a well-shaped tetradecamer, which is highlighted by the dashed red circle. Particles of this class were used to generate the refined density map of the PepQ-bound Δ526 GroEL complex at 8.3 Å resolution. Class 3 and 4 possess particle shapes consistent with an empty GroEL tetradecamer and were used to generate the refined density map of the apo Δ526 GroEL at 7.9 Å

resolution. Because of its anomalous particle shape, Class 1 was discarded. The initial refinement model employed was derived from the crystal structure of *E. coli* GroEL (PDB ID: 4HEL), blurred to a resolution of 40 Å.

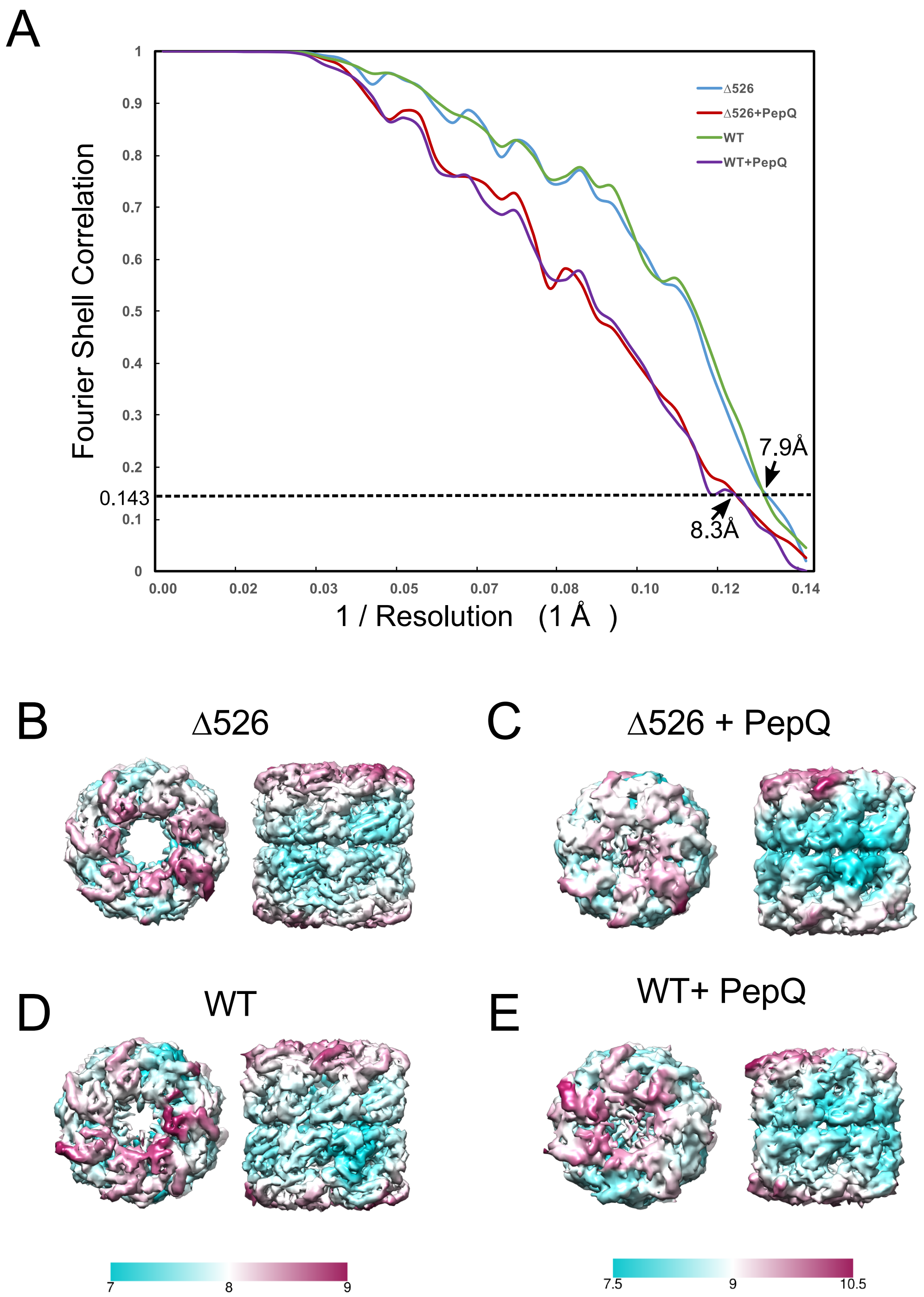

Supplementary Figure 7. Overall and local resolutions of GroEL complexes. Panel A shows the final resolution of the refined maps for all complexes determined by the gold-standard criteria and measured at 0.143 FSC. The curves for both Δ526 GroEL apo (*blue*) and PepQ-bound (*red*), as well as the wild type GroEL apo (*green*) and PepQ-

bound (*purple*), are shown. Both apo state structures have a resolution of 7.9 Å resolution, while both PepQ-bound complexes have a resolution of 8.3 Å. Panels B and D show the local resolution of the Δ526 GroEL and wild type GroEL apo structures. Panels C and E show the local resolution for the PepQ-bond Δ526 GroEL and wild type GroEL structures. In each case, the colors shown indicate the local resolution in each structure, with blue being higher resolution and pink being lower. The color map corresponding to the resolution range for each structure is shown at the bottom (in Å). In all cases, the GroEL apical domains display a lower estimated resolution (~ 9 Å), compared to the equatorial domains (~ 7 Å). The PepQ density inside chamber displayed the lowest local resolution in both maps, most likely due to the conformational heterogeneity of the non-native PepQ monomer.

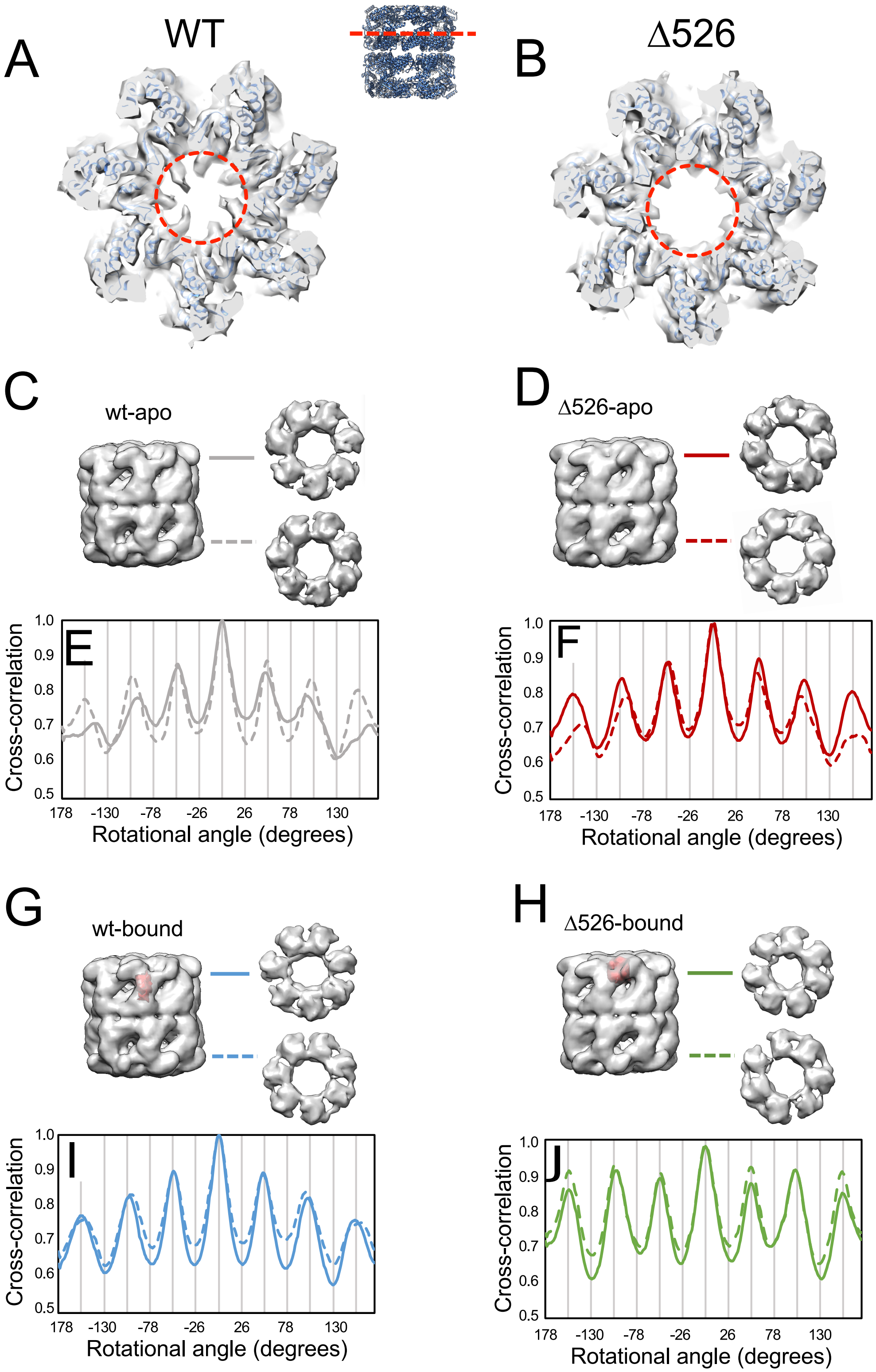

Supplementary Figure 8. Difference in C-termini densities between wild type and Δ526 GroEL, and the change of apical domain symmetry upon PepQ binding. The GroEL C-termini are partially resolved in the empty ring of the wild type GroEL complex,

but are missing in Δ526 rings. The atomic coordinates of GroEL (PDB ID: 4HEL) were fit into the density maps of wild type GroEL and Δ526. An end-on, top view of an empty wild type GroEL ring (A) and Δ526 (B) ring are shown, at the slice position indicated by the dashed red line in the inset side view. Notably, the GroEL C-termini cannot be seen in GroEL crystal structures, most likely due to their high flexibility and conformational heterogeneity. However, a substantial fraction of the C-termini are visible in the wild type GroEL structure presented here, indicated by the significant extra density visible within the dashed red circle in panel A. The observation of the C-termini in this structure is likely due to the use of C1 symmetry during model refinement. Importantly, and as expected, the same density is missing in the Δ526 ring. Rotational cross-correlation of the apical domains from both rings of wild type apo GroEL (C and E), apo Δ526 (D and F), PepQ-bound wild type GroEL (G and I) and PepQ-bound Δ526 (H and J), respectively. Solid and dashed lines indicate the two different rings of the same tetradecamer complex. The apical domains within a single ring do not show perfect 7-fold symmetry, as indicated by the differences in the height of the peaks in the rotational cross-correlation curves (E,F,I and J). However, PepQ binding induces a much larger increase in apical domain symmetry for both rings of the Δ526 tetradecamer (J).

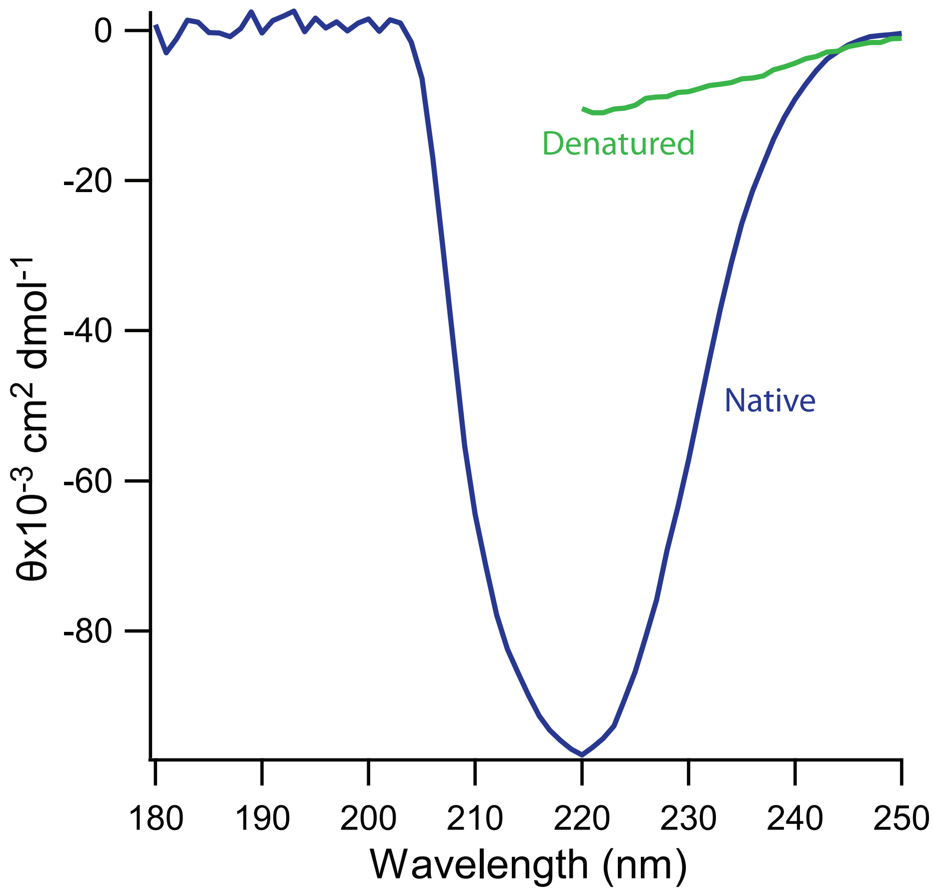

Supplementary Figure 9. Dilution of PepQ into acid-urea results in loss of observable secondary structure by circular dichroism (CD). The secondary structure of PepQ (0.1 mg/mL) was examined by far UV CD spectroscopy in native buffer (pH 7.2, *blue*) and denaturing buffer (8M Urea, pH 2.1, *green*). The CD signal of the protein in urea-containing buffer could not be acquired below 220 nm due to a dramatic increase in light scattering. Traces show the average of n = 3 independent samples.

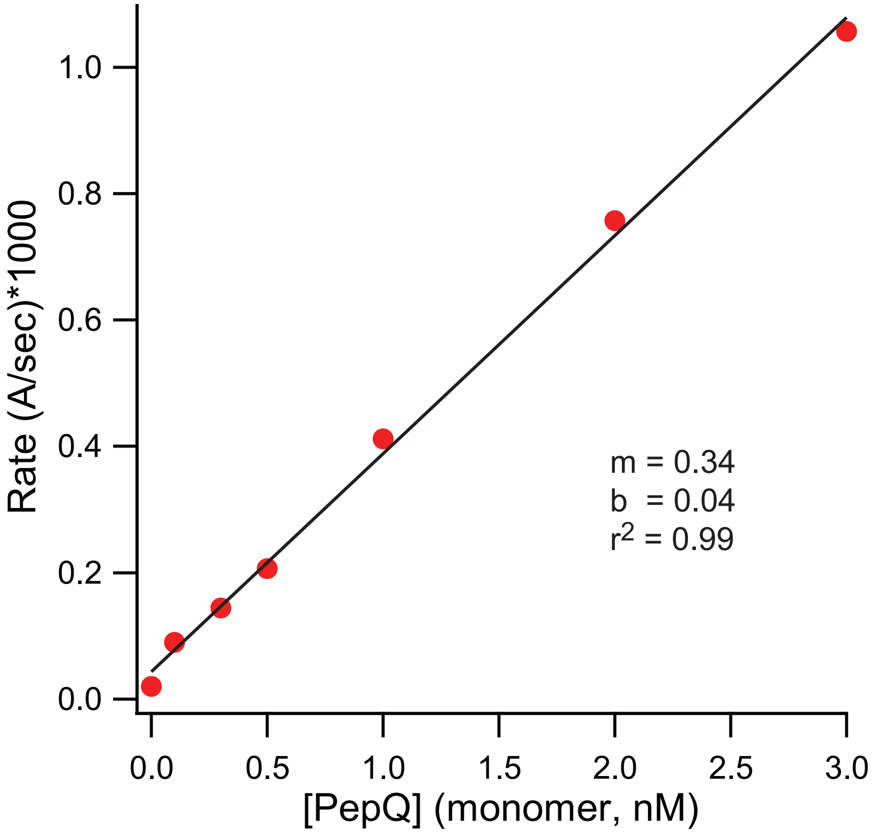

Supplementary Figure 10. Native PepQ is active at sub-nanomolar concentrations. The activity of native PepQ was measured at varying concentrations of enzyme. Values show the observed rate of a single experiment.

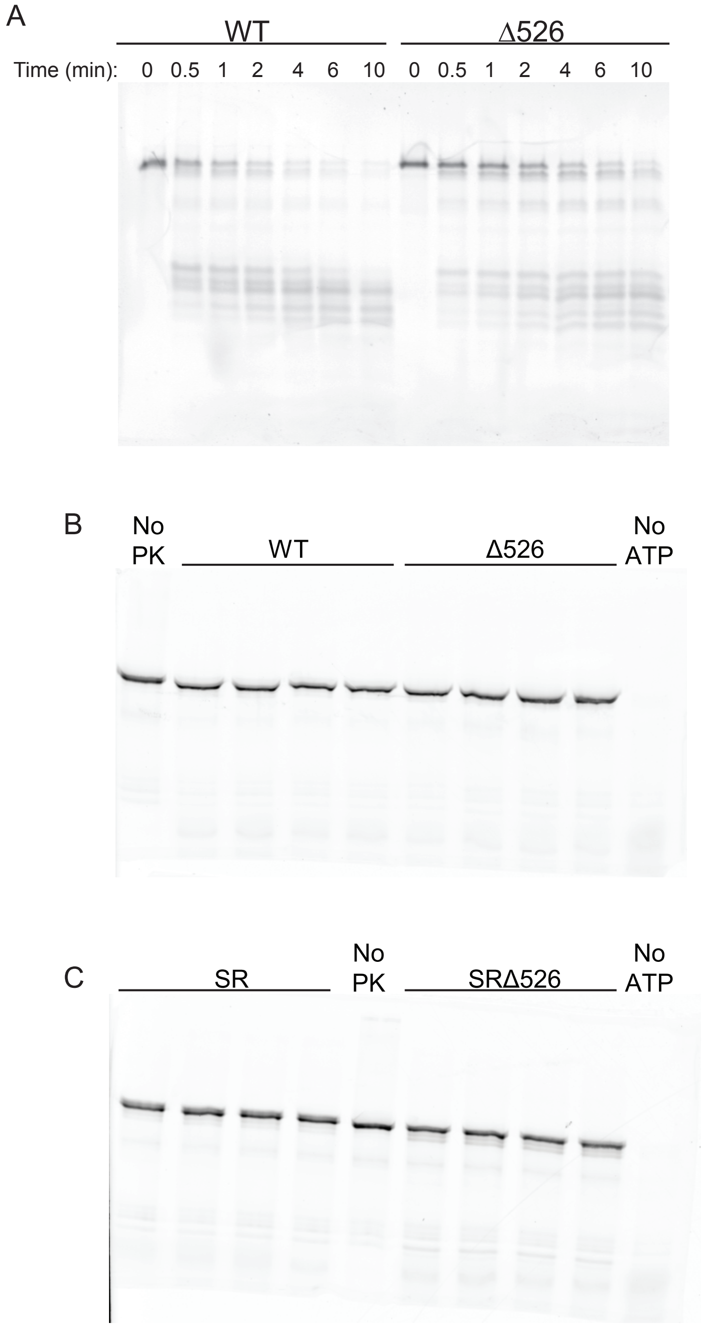

Supplementary Figure 11. Un-cropped fluorescence images of SDS-PAGE gels. (A) Example of full image of SDS-PAGE gel from Figure 5 (one replicate of three is shown). (B and C) Full images of SDS-PAGE gel from Supplemental Figure 3.

Supplementary References

1. Weaver, J., Watts, T., Li, P. & Rye, H. S. Structural basis of substrate selectivity of E. coli prolidase. *PLoS ONE* **9,** e111531 (2014).

2. Meer, B. W. V. D., Coker, G. & Chen, S. Y. S. *Resonance energy transfer : theory and data TT* . (VCH,, 1994).

3. Widengren, J. & Rigler, R. Mechanisms of photobleaching investigated by fluorescence correlation spectroscopy. *Bioimaging* **4,** 149–157 (1996).

4. Widengren, J., Rigler, R. & Mets, U. Triplet-state monitoring by fluorescence correlation spectroscopy. *J Fluoresc* **4,** 255–258 (1994).
